# Supplementary material for: Pathological Alterations in Heart Mitochondria in a Rat Model of Isoprenaline-Induced Myocardial Injury and Their Correction with Water-Soluble Taxifolin
Source: Int J Mol Sci. 2024 Oct 29;25(21):11596. doi: 10.3390/ijms252111596 (PMC11547074; doi:10.3390/ijms252111596)
Supplement: Supplementary file 1 [file ijms-25-11596-s001.zip › ijms-3277373-supplementary.pdf]

Supplementary Materials

to the manuscript of
"Pathological Alterations in Heart Mitochondria in a Rat Model of Isoprenaline-induced Myocardial Injury and Their Correction with Water-Soluble Taxifolin"

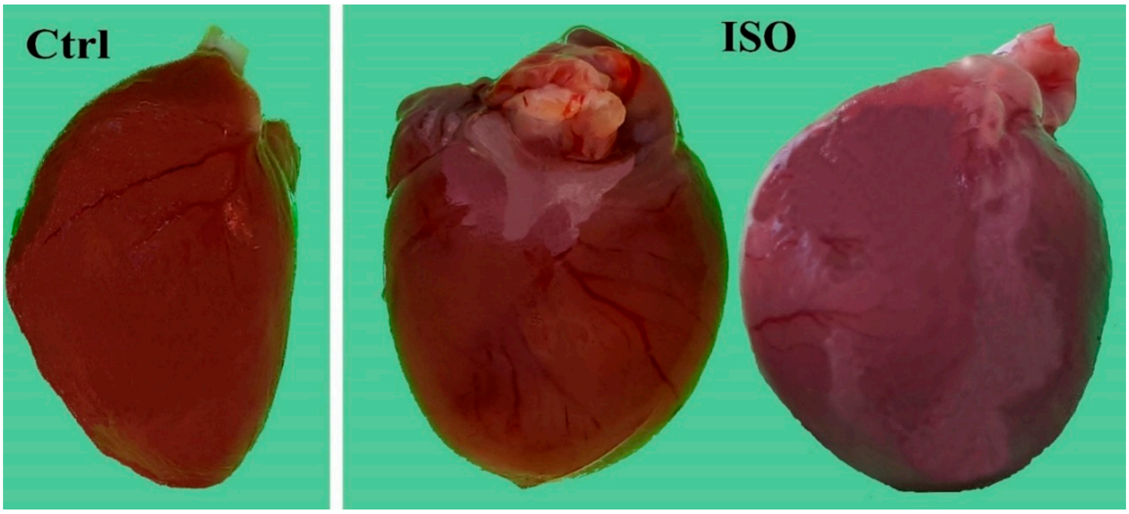

Figure S1. Representative whole hearts perfused with 1% Evans blue dye to assess myocardial injury at autopsy in the experimental groups: Ctrl – control; ISO – isoprenaline. After 14 days of double administration (24 hours apart), ISO (150 mg/kg/day) caused the formation of macroscopic regions of non-viable tissue and areas of poor perfusion. Typical changes are shown (*n* = 3). In this series of experiments, 1% percent Evan's blue was injected into the rats at a dose of 2 mL/kg before euthanasia according to the standard technique described by Houson et al., 2020 [42].
